# UNDERGRADUATE EXERCISE AND AGING STUDENTS KNOWLEDGE AND PERCEPTIONS REGARDING OLDER ADULTS

**DOI:** 10.1093/geroni/igz038.1970

**Published:** 2019-11-08

**Authors:** Tracy Davis, Michelle Zechner

**Affiliations:** 1 Rutgers University, Blackwood, New Jersey, United States; 2 Rutgers University, Scotch Plains, New Jersey, United States

## Abstract

The aging population presents far ranging implications and opportunities for individuals, families, policy makers, and healthcare providers. The U.S. Census Bureau estimates an increase in the population aged 65+, which is projected to reach 83.7 million by 2050. With this changing demographic environment, human services and health care professionals with specialized training in aging are needed, regardless of career goals all students deserve exposure to aging education. Undergraduate students have varying knowledge and attitudes towards older adults. Many times undergraduate students have had limited interactions with older adults and their attitudes and perceptions are based on interactions with grandparents and other relatives. In order to better understand undergraduate student’s knowledge and attitudes regarding older adults we surveyed a group of 50 undergraduate students enrolled in an exercise and aging course offered through the Department of Kinesiology and Health. Students were surveyed using the Facts on Aging Quiz (Breytspraak & Bandura, 2015) and the Aging Semantic Differential (ASD) (Rosencranz & McNevin, 1969). Preliminary findings suggest that students have relatively low knowledge about older adults, as the average score of the Facts on Aging Quiz was 30.4 out of 50 (SD= 3.86). However, scores on the ASD indicate that the student have generally more positive attitudes towards older adults (M=74.29; SD=20.9). At the end of the semester the same students will be surveyed again to evaluate the impact of the course. Findings from this study will be used to augment course content to increase student knowledge and attitudes about older adults.

